# GLI3 Is Required for M2 Macrophage Polarization and M2-Mediated Waldenström Macroglobulinemia Growth and Survival

**DOI:** 10.3390/ijms252313120

**Published:** 2024-12-06

**Authors:** Ava J. Boutilier, Mohammad Raad, Kailey E. Paar, Stephan J. Matissek, Cameron E. Banks, Allison L. Carl, Jenna M. Murray, Anna D. Metzler, Katja U. Koeppen, Mamta Gupta, Sherine F. Elsawa

**Affiliations:** 1 Department of Molecular, Cellular and Biomedical Sciences, University of New Hampshire, Durham, NH 03824, USA; ava.boutilier@unh.edu (A.J.B.); mohammad.raad@unh.edu (M.R.); kailey.paar@unh.edu (K.E.P.); stephan.matissek@unh.edu (S.J.M.); cameron.banks@unh.edu (C.E.B.); allison.carl@unh.edu (A.L.C.); jenna.murray@unh.edu (J.M.M.); anna.metzler@unh.edu (A.D.M.); 2 Department of Microbiology and Immunology, The Geisel School of Medicine at Dartmouth, Hanover, NH 03755, USA; 3 Department of Biochemistry and Molecular Medicine, George Washington University’s Cancer Center (GWCC), Washington, DC 20037, USA; magupta@email.gwu.edu

**Keywords:** Waldenstrom macroglobulinemia, non-Hodgkin lymphoma, tumor microenvironment, macrophage polarization, GLI protein

## Abstract

Waldenstrom macroglobulinemia (WM) is a non-Hodgkin B-cell lymphoma, characterized by bone marrow infiltration with plasma cells and lymphocytes. The tumor microenvironment (TME) plays an important role in mediating WM cell biology, but the effects of macrophages on WM biology remains unclear. Here, we investigated the effects of macrophages on WM growth and survival and identified a novel role for transcription factor GLI3 in macrophage polarization. We found that co-culture of M0 and M2 macrophages promoted WM cell growth and survival, and co-culture WM cells with M0 macrophages induced M2-like phenotypes. Interestingly, GLI3 expression was induced in M2 macrophages (not M1), leading us to perform analysis of macrophages from mice lacking Gli3 in myeloid cells (*M-Gli3^−/−^* mice). A subset of differentially expressed genes implicated a role for GLI3 in macrophage polarization. Macrophages from *M-Gli3^−/−^* mice did not induce WM cell proliferation and reduced survival compared to M2 macrophages from WT mice. In addition, in vitro polarization of M0 macrophages from *M-Gli3^−/−^* was not able to induce M2 markers such as CD163, despite inducing iNos expression (M1 marker). Taken together, these results suggest a role for M2 macrophages in promoting WM cell growth and identify GLI3 as a modulator of macrophage polarization.

## 1. Introduction

Waldenstrom macroglobulinemia (WM) is an indolent B-cell malignancy characterized by the infiltration of the bone marrow with plasma cells, plasmacytoid lymphocytes, and small lymphocytes [1,2,3,4]. WM may also be known as a lymphoplasmacytic lymphoma (LPL) with an immunoglobulin M (IgM) paraprotein by the World Health Organization [1,5]. There is no standard treatment for WM and treatment programs are variable. As of 2024, only two FDA-approved treatments exist, ibrutinib and zanubrutinib, and no cure has been discovered to date [6,7]. Around three-quarters of WM patients present with symptoms at time of diagnosis, and cytopenia and anemia is common as the bone marrow tumor burden increases [2].

The development and progression of B-cell malignancies involve the interaction between malignant B-cells and non-tumoral cells within the TME. Several studies have shown that the TME modulates WM cell biology [8,9,10] and can elicit pro-proliferative states in WM cells. While the full function of the TME in the progression of WM has not been described, some efforts have been made to quantify the importance of cells in the TME in WM [2,8,9,10,11,12,13,14,15,16].

The bone marrow is made up of both immune and non-immune cells [14]. The full function and mechanism of these infiltrating cells in WM is limited. Mast cells and T-cells have been shown to contribute to WM progression through CD40L/CD40 interactions [15] and expression of PD-1 and PD-L1/L2, respectively, contributing to both growth and immune suppression [12,17]. Similarly, mesenchymal stem cells and bone marrow stromal cells (BMSC) have been shown to play an important role in normal and malignant cell biology [18,19].

Previously, we have shown that mantle cell lymphoma (MCL, a type of B-cell lymphoma) TME is enriched with tumor-associated macrophages and promotes macrophage polarization toward an M2 phenotype, which induces MCL progression in vivo [20]. More recently, we have identified CCR1 as a key driver of macrophage programming in MCL and induction of a pro-tumor TME and, consequently, MCL progression [21]. The role of macrophages in the WM TME has not yet been investigated. With this background, we examined the critical role of macrophages in WM growth and identify GLI3 as a key modulator of macrophage programming.

## 2. Results

### 2.1. M0 and M2 Macrophages Increase WM Cell Growth and Survival

**Figure 1:**
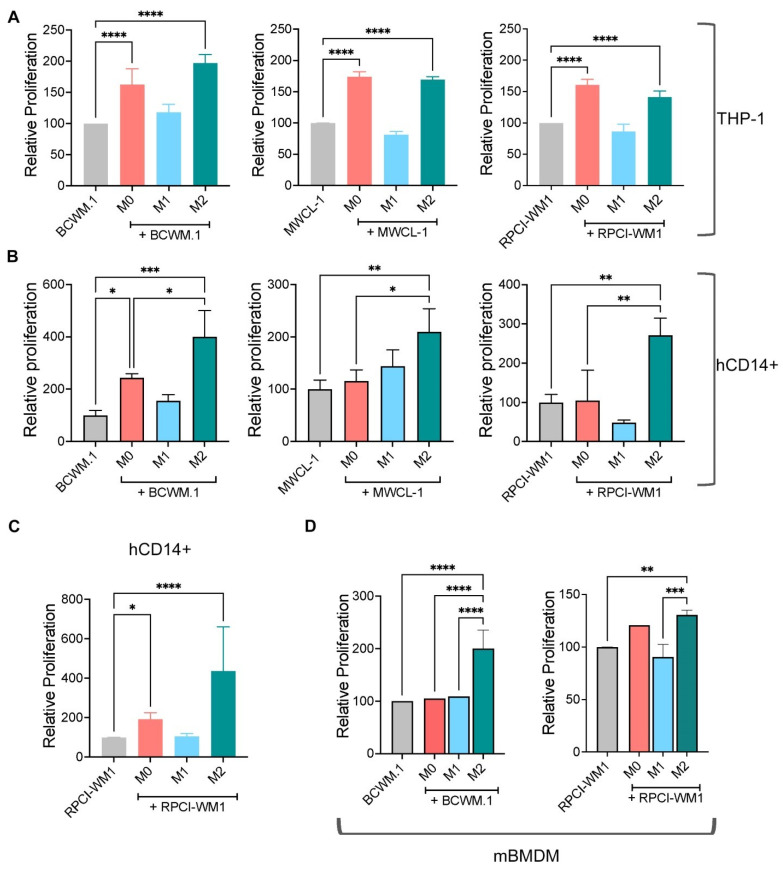
M0 and M2 macrophages promote WM cell proliferation. (**A**) M0, M1, or M2 macrophages derived from THP-1 human monocytes were co-cultured with WM cell lines (BCWM.1, MWCL-1, or RPCI-WM1) and relative proliferation of WM cells was measured via XTT assay after 72 h of co-culture. (**B**) CD14^+^ cells were isolated from human PBMCs, then differentiated into M0, M1, or M2 macrophages. Macrophages from different donors (left to right: donor 16, 18, 19) were co-cultured with WM cells for 72 h followed by assessment of WM cell proliferation using XTT assay. (**C**) Relative proliferation of RPCI-WM1 cells co-cultured with macrophages from different donors (*n* = 7). (**D**) Bone marrow-derived macrophages were generated from C57BL/6J mice (*n* = 4), as described in Section 4, then differentiated into M0, M1, or M2 macrophages. Macrophages were co-cultured with BCWM.1 or RPCI-WM1 cells for 72 h followed by XTT assay to determine cell proliferation. These experiments were repeated at least 3 times, and the results are presented as the average of 3 independent experiments, each performed in triplicate wells ± SEM. * *p* < 0.05; ** *p* < 0.01; *** *p* < 0.001; **** *p* < 0.0001.

M2 macrophages have demonstrated pro-tumorigenic effects in several malignancies [17]; however the biological role of macrophage populations in WM has not been investigated. To determine if M1 or M2 macrophages play a role in WM biology, we first generated M0, M1, and M2 macrophages from the THP-1 human monocyte cell line and co-cultured them in direct contact with the three WM cell lines, BCWM.1, MWCL-1, and RPCI-WM1. We found that M2 macrophages increased the proliferation of WM cells. Interestingly, M0 macrophages also induced the proliferation of WM cells (Figure 1A). However, co-culture of WM cells with M1 macrophages had no effect on WM cell proliferation. We confirmed the THP-1 results using primary human CD14^+^ cells to generate macrophages. Similar results to THP-1 cells were obtained using CD14^+^-derived M2 macrophages from different donors where they promoted a significant increase in WM cell growth (Figure 1B). We also found that M0 macrophages from different healthy donors increased RPCI-WM1 cell growth (Figure 1C). Finally, M2 cells from murine BMDM also increased WM cell growth (Figure 1D).

**Figure 2:**
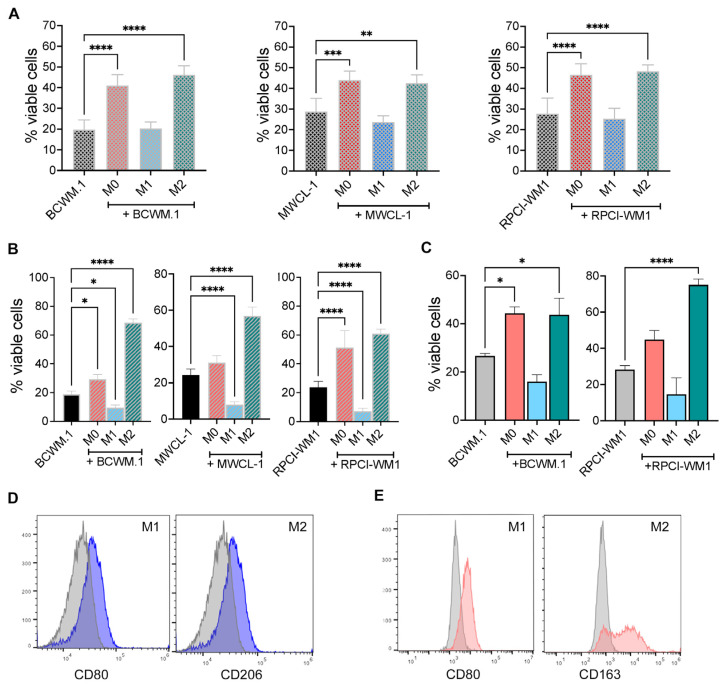
M0 and M2 macrophages promote WM cell survival. (**A**) Viability of WM cells was measured via Trypan blue exclusion after 72 h of co-culture with THP-1-derived M0, M1, or M2 macrophages. (**B**) WM cell viability after 72 h of co-culture with CD14^+^ cell-derived M0, M1, or M2 phenotypes from PBMC donors (*n* = 3 donors). (**C**) Viability of WM cell lines was measured after 72 h of co-culture with M0, M1, or M2 macrophages from C57BL/6 mice (*n* = 3 mice). These experiments were repeated at least 3 times, and the results are presented as the average of 3 independent experiments, each performed in triplicate wells ± SEM. (**D**) Flow cytometry analysis of human CD14^+^ cell-derived cells. M1 and M2 cells were derived as described in Section 4 and harvested and stained with CD80 (M1) or CD206 (M2) antibodies. Cells were gated on viable cells. (**E**) Flow cytometry analysis of mouse BMDM. M1 and M2 cells were derived as described in Section 4 and then stained with CD80 (M1) or CD163 (M2) antibodies. Gating was carried out on the viable cell population. Gray histograms (Isotype controls); Colored histograms (indicated markers), * *p* < 0.05; ** *p* < 0.01; *** *p* < 0.001; **** *p* < 0.0001.

Next, we examined the effect of macrophages on WM cell survival. Similar to the effects on cell proliferation, WM cells cultured in the presence of M0 or M2 macrophages derived from THP-1 cells had increased survival compared with WM cells cultured alone (Figure 2A). Similarly, M0 and M2 macrophages derived from peripheral blood of healthy donors increased WM cell survival (Figure 2B), and M0 and M2 BMDM from mice also increased WM cell survival (Figure 2C). As we have previously shown using human cells [20], THP-1 and PBMC-derived M1 macrophages express CCL2 and IL-1*b* and CD80 (Figure 2D and Figure S1A–C), and M2 macrophages express CCL22 and CD206 (Figure 2D and Figure S1A–C). Similarly, mouse M1 cells from BMDM express CD80 and M2 cells express CD163 (Figure 2E and Figure S1D). Taken together, these results show that M0 and M2 macrophages promote WM cell growth and survival.

### 2.2. WM Cells Promote an Inhibitory M2 Macrophage Phenotype

**Figure 3:**
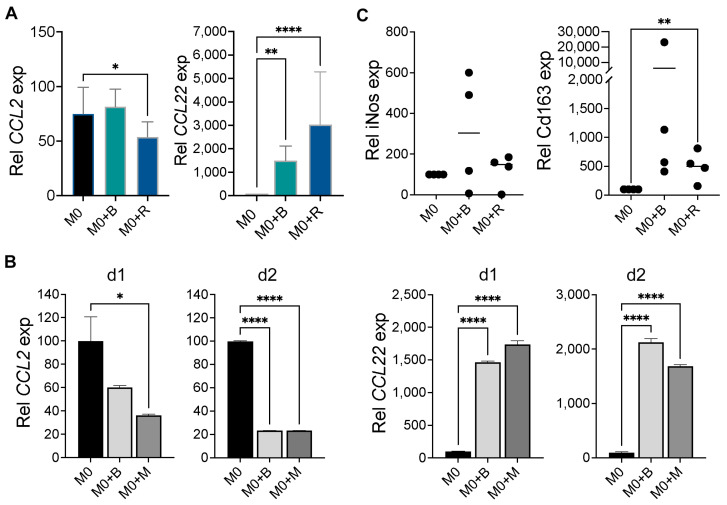
WM cells induce an inhibitory M2 macrophage phenotype. (**A**) M0 macrophages from THP-1 cells (*n* = 4) were co-cultured with BCWM.1 (B) or RPCI-WM1 (R) cells for 72 h. RNA was isolated and used to determine CCL2 (M1) and CCL22 (M2) gene expression by RT-qPCR. (**B**) M0 macrophages from CD14^+^ cells from PBMCs (*n* = 2 donors: d1 and d2) were co-cultured with BCWM.1 (B) or MWCL-1 (M) cells for 72 h followed by RT-qPCR to determine *CCL2* (M1) and *CCL22* (M2) gene expression. (**C**) BMDM (M0) from C57BL6/J mice (*n* = 4 mice) were co-cultured with BCWM.1 (B) or RPCI-WM1 (R) cells for 72 h followed by RT-qPCR to determine *iNos* (M1) and *Cd163* (M2) gene expression. Results are presented as data generated by macrophages from individual mice, and the bars represent mean values. * *p* < 0.05; ** *p* < 0.01; **** *p* < 0.0001.

The observation that M0 macrophages promoted WM cell growth and survival (Figure 1 and Figure 2) led us to investigate the possibility that WM cells may modulate macrophage phenotype. To address this, we co-cultured WM cells with M0 macrophages from THP-1, CD14^+^ cells from PBMCs, and murine BMDM. We found that co-culture of WM cells with THP-1-derived M0 macrophages induced the expression of CCL22, an M2 marker with no change or slight decrease in the M1 gene CCL2 (Figure 3A). Similarly, when WM cells were co-cultured with CD14^+^-derived M0 macrophages, there was a significant increase in CCL22 expression and a decrease in the M1 gene CCL2 (Figure 3B). Finally, WM cells co-cultured with murine BMDM M0 macrophages induced an increase in the M2 gene Cd163 expression but not in the M1 gene iNos (Figure 3C). This suggests that WM cells promote the differentiation of macrophages into an inhibitory M2 phenotype, which in turn promotes their growth and survival.

### 2.3. GLI3 Expression Is Induced in M2 Macrophages

**Figure 4:**
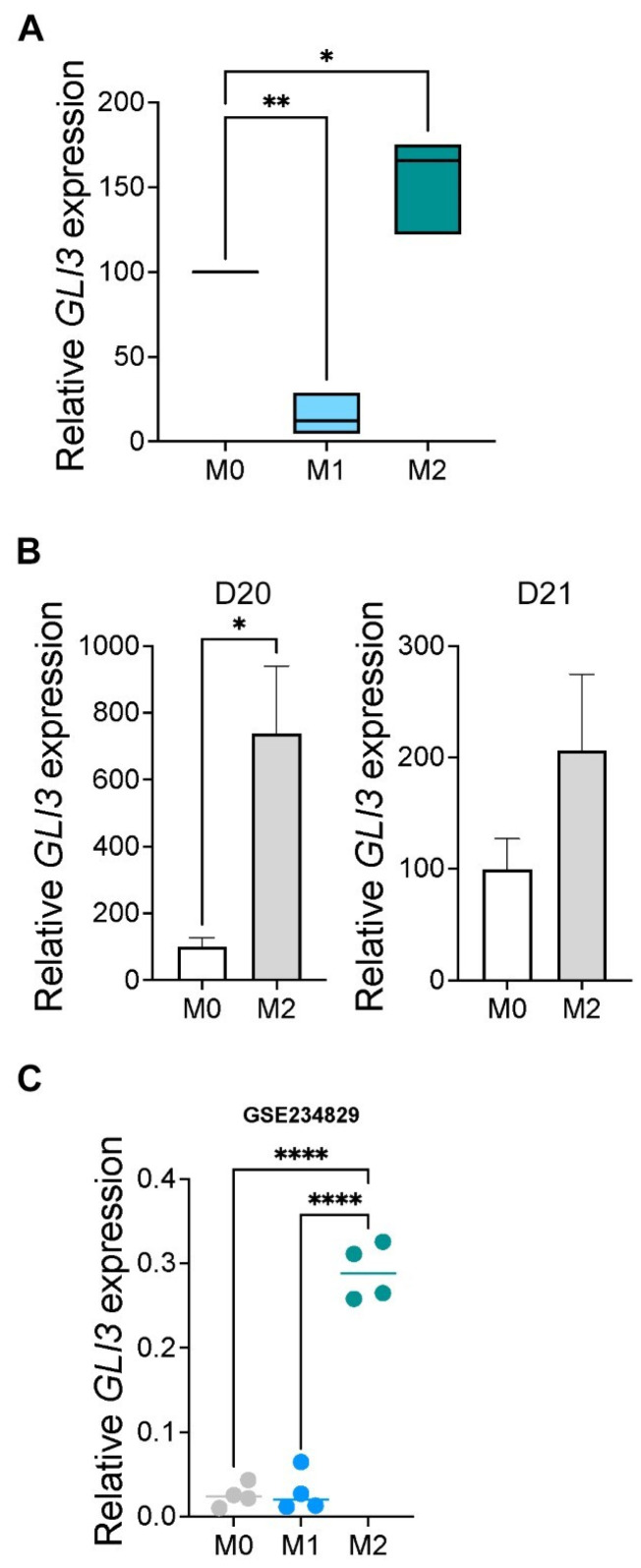
GLI3 expression is induced during in vitro M2 macrophage polarization. (**A**) M0, M1, and M2 macrophages were derived from THP-1 cells as described in Section 4 and GLI3 expression was determined by RT-qPCR. This experiment was repeated 3 times, and data are presented as the average of 3 experiments, each performed in triplicate ± SEM. (**B**) M0 and M2 macrophages were derived from CD14^+^ cells isolated by negative selection from PBMCs (D20: donor 20; D21: donor 21). RNA was isolated and used to determine GLI3 expression by RT-qPCR. Data are presented as triplicate wells for cells generated from each donor. (**C**) GLI3 expression was examined in RNA-seq data using THP-1-derived M0, M1, and M2 (GSE234829). * *p* < 0.05; ** *p* < 0.01; **** *p* < 0.0001.

In previous work, we have shown that the transcription factor GLI3 mediates TLR-induced cytokine responses in monocytes [22]. Therefore, we investigated the expression of GLI3 in human M1 and M2 macrophages compared with M0 macrophages. We generated M0, M1, and M2 macrophages from THP-1 cells, as described earlier, then used them to examine GLI3 expression by RT-qPCR. We found that GLI3 expression was significantly increased in M2 macrophages compared with M0 macrophages (Figure 4A). Similarly, using CD14^+^-derived macrophages, we found that GLI3 expression was significantly increased (Figure 4B).

Finally, using public genome-wide data (GSE234829), we identified GLI3 expression in M0, M1, and M2 wild-type macrophages and found a significant increase in GLI3 expression in M2 macrophages compared with M0 (resting macrophages) (Figure 4C). These studies show that GLI3 is induced in M2 macrophages and suggest GLI3 may modulate M2 macrophage biology.

### 2.4. GLI3 Promotes M2 Macrophage Polarization

**Figure 5:**
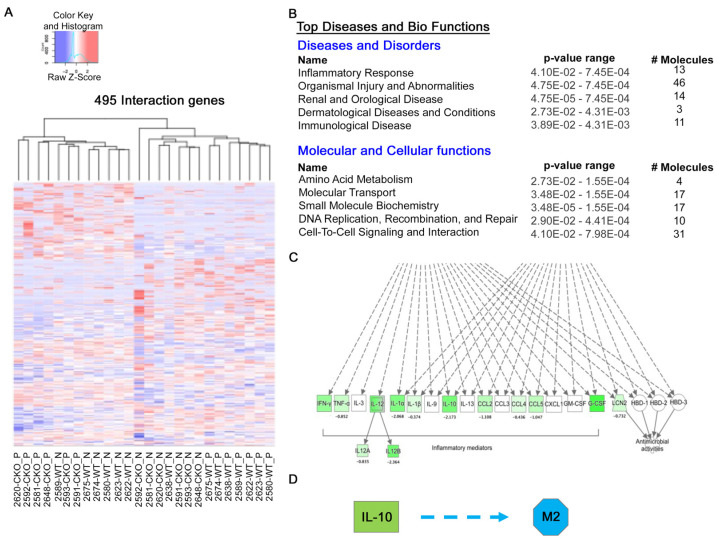
RNA-seq identifies cytokines as targets of GLI3. RNA-seq on macrophages derived from *WT* and *M-Gli3^−/−^* mice (*n* = 4/group) treated with LPS or DPBS. (**A**) Heatmap of genes with significant interaction effects between genotype and treatment. Rows are scaled by Z-score, with shades of blue indicating values below the mean for each row (gene) and shades of red indicating values above the row mean. Samples in the columns are hierarchically clustered by Euclidean distance. (**B**) Ingenuity pathway analysis (IPA) of significant interaction genes identified the top 5 pathways in the “diseases and disorders” category and in the “molecular and cellular functions” category. (**C**) IPA analysis of genes that were significantly differentially expressed between *M-Gli3^−/−^* and *WT* mice revealed broad downregulation of inflammatory mediators in *M-Gli3^−/−^* mice. Green shading indicates the extent of downregulation in *M-Gli3^−/−^* versus *WT* mice. (**D**) IPA predicts inhibition of M2 polarization in CKO mice (activation Z-score = −1.4, *p* = 2.2 × 10^−3^) based on downregulation of polarization activator IL-10, among other genes.

Because GLI3 expression is induced in M2 macrophages, we analyzed RNA-seq data from macrophages stimulated with lipopolysaccharide (LPS) (PRJNA1071120). For this study, Incomplete Freund’s Adjuvant (IFA)-elicited peritoneal macrophages from *M-Gli3^−/−^* or WT mice [22] were treated with 100 ng/mL LPS or left untreated for 1 h, and RNA was subjected to RNA-sequencing. Using a generalized linear model in edgeR, we identified 495 genes with significant interaction effects between genotype and LPS treatment stimulation (Figure 5A). For genes with a significant interaction between genotype and treatment, untreated macrophages from *M-Gli3^−/−^* mice clustered with LPS-treated macrophages from WT mice, suggesting that Gli3 knockout antagonizes the LPS response of these genes. Ingenuity pathway analysis of these genes revealed several pathways that were enriched in these significant interaction genes, including “inflammatory response” and “immunological disease” (Figure 5B). This analysis also identified “cell-to-cell signaling and interaction” as one of the top pathways in the “molecular and cellular functions” category to be affected by GLI3 in response to LPS stimulation (Figure 5B).

Our analysis identified 1147 genes that were significantly differentially expressed (*p* < 0.05 and absolute log2 fold change > 1) in macrophages from *M-Gli3^−/−^* and WT mice, and “differential regulation of cytokine production” was one of the top five significant canonical pathways (*p* = 8.3 × 10^−3^) identified by IPA (Figure 5C). Cytokines such as CCL2, IL-10, IL-1a and IL-1*b*, and TNF-*a* were among the genes that showed a broad downregulation in *M-Gli3^−/−^* mice (Figure 5C). Analysis also suggested GLI3 may play a role in M2 macrophage polarization, as IPA predicts inhibition of M2 polarization in macrophages from *M-Gli3^−/−^* mice (activation Z-score = −1.4, *p* = 2.2 × 10^−3^) based on upregulation of the M2 polarization inhibitors THRA and PDK1 and downregulation of polarization activators IL-10 and CYBB (Figure 5D). Therefore, these data, along with data in Figure 4, suggest a role for GLI3 in M2 macrophage polarization.

### 2.5. GLI3 Is Required for Macrophage-Mediated Effects on WM Cells

**Figure 6:**
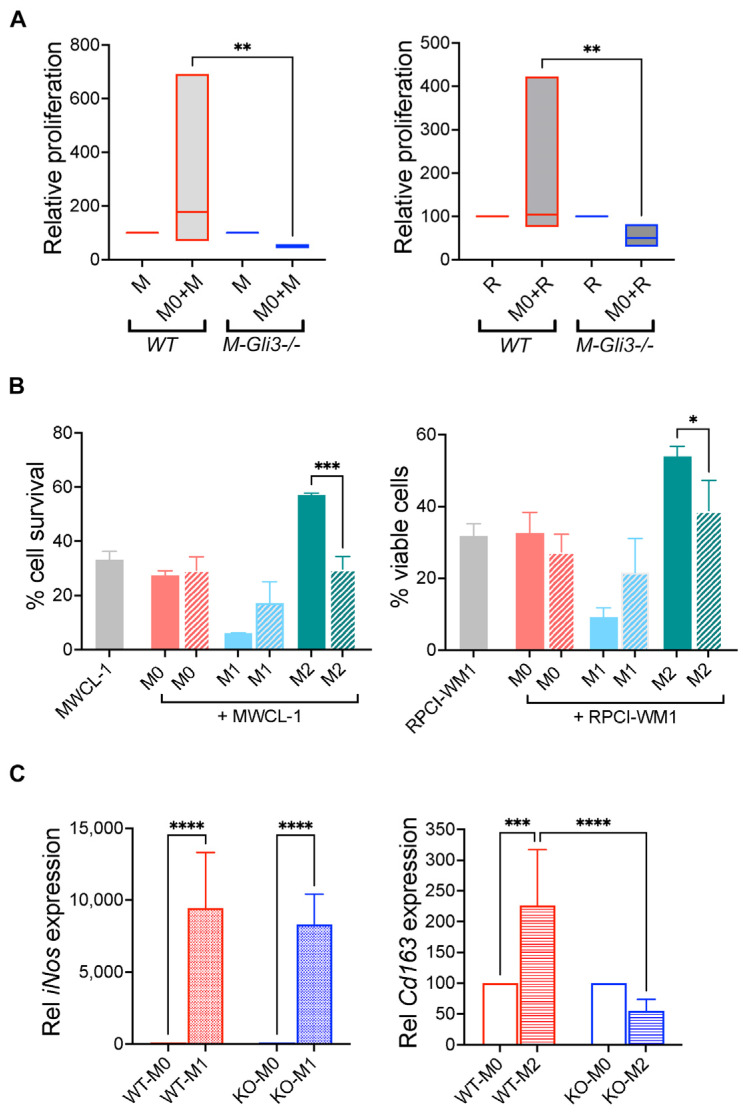
GLI3 is required for M2 macrophage polarization and downstream effects on WM cell growth and survival. (**A**) Bone marrow-derived M0 macrophages were generated from wild-type (*WT*; *n* = 4) and *M-Gli3^−/−^* (*n* = 3) mice and co-cultured with MWCL-1 (M) or RPCI-WM1 (R) cells for 72 h followed by determination of WM cell proliferation by XTT assay. (**B**) Bone marrow-derived M0, M1, and M2 macrophages were generated from WT (*n* = 2; solid bars) and *M-Gli3^−/−^* (*n* = 4; striped bars) mice and co-cultured with MWCL-1 (M) or RPCI-WM1 (R) cells for 72 h followed by determination of WM cell viability by trypan blue exclusion. (**C**) Expression of M1 (iNos) and M2 (Cd163) macrophage markers was examined in M0, M1, and M2 macrophages from *WT* (*n* = 3) and *M-Gli3^−/−^* (*n* = 3) mice. Results are presented as mean values ± SEM. * *p* < 0.05; ** *p* < 0.01; *** *p* < 0.001; **** *p* < 0.0001.

To address the possibility that GLI3 plays a role in the biological function of macrophages on WM cells, BMDM were generated from *M-Gli3^−/−^* and *WT* mice and M0 macrophages were co-cultured with WM cell lines MWCL-1 and RPCI-WM1. We found that M- *M-Gli3^−/−^* M0 macrophages were unable to increase the proliferation of WM cells, while *WT* M2 macrophages increased WM cell proliferation (Figure 6A). To address the possibility that GLI3 may play a role in macrophage-dependent WM cell viability, we performed a direct co-culture of bone marrow-derived macrophages isolated from *M-Gli3^−/−^* and *WT* mice and found that M2 macrophages from only *WT* mice were able to increase the viability of WM cells (Figure 6B). There was a significant reduction in WM cell viability in co-culture with M2 macrophages from *WT* and *M-Gli3^−/−^* mice (Figure 6B). We performed qPCR on macrophages from *M-Gli3^−/−^* and *WT* mice that were polarized to M0, M1, and M2 phenotypes and found that WT macrophages were able to express both M1 and M2 macrophage genes, as indicated by expression of the M1 marker iNos and the M2 marker Cd163, while *M-Gli3^−/−^* were only able to express the M1 gene iNos but not the M2 gene Cd163 compared with macrophages from WT mice, indicating that Gli3 is required for successful M2 polarization (Figure 6C). Taken together, these results suggest that GLI3 is exclusively required for macrophage polarization to M2 phenotype and, consequently, macrophage-mediated biological effects on WM cells.

## 3. Discussion

The tumor microenvironment plays an important role in solid tumors as well as hematologic malignancies [23,24,25,26,27]. Studies have shown that the TME plays a role in WM biology [2,8,9,10,12,15,16,19,28,29,30,31]. BMSCs have been shown to regulate the proliferation of WM cells while contributing to increased drug therapy resistance [29,30,31]. Mast cells have also been shown to promote WM cell growth and survival [15]. We have shown increased infiltration of innate immune cells such as macrophages in the TME of Mantle cell lymphoma (MCL) in a syngeneic mouse model [18]. A recent study reported increased CD163-expressing macrophages in the WM TME [32], thus setting the platform for macrophage infiltration in WM. We and others have reported that macrophages exist in two biological states in tumors, the classical M1 and alternative M2 macrophages with either anti-tumor or pro-tumor functions [17,33,34,35,36,37,38,39,40]. M2 macrophages have been shown to promote cancer growth and are associated with poor survival in various cancers [17,18,34,36,37,38]. Furthermore, an increased M1/M2 TAM ratio has been linked to an improved 5-year prognosis in several cancers [35,36,37,38,39,40]. However, studies of the role of M2 macrophages in WM are lacking. Our data show that in vitro and ex vivo polarized M2 macrophages, but not M1 macrophages, increase the growth and viability of WM cells (Figure 1 and Figure 2). This is consistent with previous studies showing a role for M2 in other cancers [17,18,35,36,37,40,41]. Our data also show that WM cells influence macrophage polarization by inducing them toward an M2 phenotype (Figure 3).

To identify the mechanism responsible for the in vitro and/or ex vivo macrophage polarization, we focused on the transcription factor GLI3. GLI3 is a member of the hedgehog (HH) signaling pathway, where it plays a role in embryonic development and in cancer [42]. We have recently shown that GLI3 plays a role in macrophage biology, where it modulates the inflammatory response to toll-like receptor (TLR) stimulation [22]. Here, we show that GLI3 is highly upregulated in M2 macrophages and plays a role in M2 macrophage polarization and subsequent pro-tumorigenic effects of M2 cells (Figure 4, Figure 5 and Figure 6). While the effects of GLI3 on macrophage differentiation have not been reported prior to this study, studies have suggested that one of the main immunosuppressive roles of the HH pathway is the polarization of macrophages toward the M2 phenotype, where in breast cancer, M2 macrophage polarization was reduced upon inhibition of HH signaling [42].

We identified a novel role for GLI3 in M2 macrophage polarization, as macrophages from mice lacking Gli3 in myeloid cells (*M-Gli3^−/−^*) [22] were unable to increase the proliferation or viability of WM cells or polarize M0 cells to an M2 phenotype (Figure 5 and Figure 6). This is supported by previous studies that reported an increase in CD163-expressing macrophages in WM patients [32]. Additionally, stimulation of murine macrophages from *M-Gli3^−/−^* mice resulted in downregulation of inflammatory gene signatures and the M2 macrophage-specific cytokine IL-10 compared with macrophages from wild-type (WT) mice (Figure 5). While these data support a role for GLI3 in inflammation, they also suggest GLI3 can mediate anti-inflammatory responses in the TME by promoting an M2 anti-inflammatory cell. Future studies to delineate the inflammatory/anti-inflammatory roles of GLI3 will enhance our understanding of the multiple roles of GLI transcription factors in immune cells, and these studies are necessary in order to target GLI3 to regulate the TME in WM.

In summary, we provide evidence that M2 macrophages promote WM cell growth and survival via the transcription factor GLI3. Our results, paired with additional studies to understand the mechanisms behind macrophage-dependent WM cell growth and survival, may lead to the development of novel therapies that benefit WM patients.

## 4. Materials and Methods

### 4.1. Cell Lines and Primary Cells

BCWM.1, MWCL-1, and RPCI-WM1 Waldenström macroglobulinemia cell lines have been previously described [43,44,45]. The cells were maintained in RPMI 1640 supplemented with 10% FBS and 1% antibiotic-antimycotic (AA), and the cells were passaged every 3–4 days. THP-1 cells were purchased from ATCC (Manassas, VA, USA) and cultured in RPMI 1640 supplemented with 10% FBS, 1% AA, and 0.05 mM b-Mercaptoethanol.

Primary human monocytes were purified from peripheral blood mononuclear cells (PBMCs) obtained from leukoreduction cones from healthy donors from the Oklahoma Blood Institute (Oklahoma City, OK, USA). Cells were separated on a Ficoll-hypaque gradient as previously published [10,22] to isolate mononuclear cells. Monocytes were isolated from total PBMCs by magnetic cell sorting using the EasySep human buffy coat CD14^+^ negative selection kit (Stemcell Technologies, Seattle, WA, USA) according to the manufacturer’s protocol.

### 4.2. Mouse Bone Marrow-Derived Macrophages (BMDMs)

C57BL/6J mice (Jackson Laboratory stock #: 000664) were obtained from the Jackson laboratory (Bar Harbor, ME, USA) and bred at the Animal Resource Office (ARO) at the University of New Hampshire. BMDMs were generated from C57BL/6J mice as previously published [22]. Briefly, bone marrow was flushed from mouse femurs and tibias and was plated in RPMI 1640 supplemented with 10% FBS, 1% antibiotic-antimycotic (AA) plus 15% L929 media to generate BMDMs. Media was replenished every 3 days, and on day 7, macrophages were used in experiments as described or differentiated into M0, M1, or M2.

GLI3-deficient macrophages were obtained from mice with Gli3 knockout in myeloid cells (*M-Gli3^−/−^*) or wild-type (WT) littermates as previously described [22]. Bone marrow-derived macrophages were generated similar to C57BL/6J mice. All animal studies were approved by the UNH Institutional Animal Care and Use Committee.

### 4.3. Macrophage Differentiation/Polarization

To differentiate the human THP-1 monocyte cell line into macrophages, 50 ng/mL phorbol 12-myristate 13-acetate (PMA) was added to cell culture media and incubated for 24 h. After 24 h, the cells were washed with complete media to remove PMA and allowed to rest for 24 h to generate M0 macrophages. M0 macrophages were then differentiated into M1 and M2 phenotypes using 10 ng/mL lipopolysaccharide (LPS) and 100 ng/mL interferon-gamma (IFN-γ) for M1, and 20 ng/mL recombinant human interleukin-4 (IL-4) and 20 ng/mL recombinant human interleukin-13 (IL-13) for M2.

For CD14^+^ differentiation, cells were cultured in the presence of recombinant human M-CSF (50 ng/mL) for 7 days, with media supplemented every 2–3 days. After 7 days, M0 macrophages were washed to remove M-CSF and then differentiated into M1 or M2 as described for THP-1 cells.

For mouse BMDM differentiation, macrophages were used on day 7 culture with L929 media, washed, and then differentiated into M1 macrophages using 1 µg/mL LPS and 300 ng/mL IFN-γ, or M2 macrophages using 40 ng/mL IL-10 and 40 ng/mL IL-13. All recombinant proteins were purchased from Peprotech (Cranbury, NJ, USA).

### 4.4. RNA Isolation and Reverse Transcription Quantitative PCR (RT-qPCR)

Total RNA was extracted using TRIsure reagent (Bioline, London, UK), and cDNA was synthesized using Promega Moloney Murine Leukemia Virus (M-MLV) reverse transcriptase as previously described [8,9,22,46]. Reverse transcription quantitative PCR (RT-qPCR) was performed as previously reported [25,47]. The primers used were purchased from Integrated DNA Technologies (IDT-DNA, Coralville, IA, USA), and primer sequences are provided in Table 1.

### 4.5. Assessment of Cell Proliferation

Cell proliferation was evaluated using TACS XTT Cell Proliferation Assay (R&D Systems, Minneapolis, MN, USA). CD14^+^ and human THP-1 monocytes were seeded in 24-well plates as described above and polarized into M0, M1, and M2 phenotypes. WM cells (0.5 × 10^6^ cells/well) were added to macrophages for 3 days. After 3 days of co-culture, WM cells were removed from each well and re-plated in a new, 48-well plate. XTT reagent was then added at a 1:50 ratio and incubated at 37 °C for 3 h. After 3 h, absorbance was measured using a SpectraMax M2e microplate reader (Molecular Devices, San Jose, CA, USA) at 490 nm with reference at 630 nm.

### 4.6. Assessment of Cell Viability

Cell viability was determined using trypan blue exclusion. Trypan blue was purchased from ThermoFisher Scientific (Waltham, MA, USA). Cells were counted using a Luna II automated cell counter (Logos Biosystems, Annandale, VA, USA). CD14^+^ or human THP-1 monocytes (0.5 × 10^6^ cells/well) were differentiated into M0, M1, and M2 phenotypes (adherent cells) and then co-cultured with WM cell lines (non-adherent cells; 0.5 × 10^6^ cells/well) in 24-well plates for 3 days followed by determination of WM cell viability (non-adherent cells only).

### 4.7. Flow Cytometry

To detect human and mouse M1 and M2 markers, 0.5 × 10^6^ cells were washed in FACS buffer (phosphate-buffered saline (PBS) containing 2% FBS and 0.05% sodium azide) and incubated with fluorochrome-conjugated antibodies or isotype controls for 20 min at 4 °C. Cells were washed and resuspended in 300 mL FACS buffer, and data acquisition was carried out on a BD Accuri C6 flow cytometer. For human CD14^+^ cells, CD80 was used for M1 and CD206 was used for M2 cells. For mouse BMDM, CD80 was used for M1 and CD163 was used for M2 cells. All antibodies were purchased from BD Biosciences (Franklin Lakes, NJ, USA). Gating was carried out on the viable population based on size exclusion using forward and side scatter. Analysis was performed using FlowJo Software v10.7.1 (Ashland, OR, USA).

### 4.8. RNA-Sequencing

RNA-sequencing was performed using macrophages derived from WT and *M-Gli3^−/−^* mice. Mice received an i.p. injection of incomplete Freund’s Adjuvant. After 3 days, macrophages were harvested by peritoneal lavage. One million macrophages were plated per well in 1 mL of media (IMDM+10% FBS+1% A/A) and allowed to adhere for 1 h. Cells were washed and treated with 100 ng/mL LPS or left untreated for 1 h. Cells were harvested using TRIsure for RNA isolation as described above. RNA sequencing was performed at the Hubbard Center for Genome Studies (University of New Hampshire). RNASeq libraries were prepared using KAPA/Roche mRNA HyperPrep Kit (KAPA/Roche, 8098123702; Roche, Indianapolis, IN, USA) per the manufacturer’s instructions. The product was purified using KAPA Pure Beads (KAPA/Roche, 7983298001; Roche, Indianapolis, IN, USA). The purified product was then amplified, bead purified, and finally, eluted in 20 µL of 10 mM Tris-Cl, pH 8.5 (Qiagen, 19086; Germantown, MD, USA). Libraries were then quantified with a dsDNA HS Assay Kit (ThermoFisher Scientific, Waltham, MA, USA; Q32854) and validated using a TapeStation 4200 (Agilent Technologies, Inc., Santa Clara, CA, USA; G2991BA) with a High Sensitivity D5000 ScreenTape and associated reagents (Agilent, 5067-5592 [48,49]). Samples were sequenced on a HiSeq 2500 instrument (Illumina, San Diego, CA, USA) using a rapid paired-end flow cell v2. Forward and reverse read lengths were 250 base pairs, and indexing reads were dual 8-mers. The data were demultiplexed using Illumina bcl2fastq v2.20.0.422.

Sequencing reads were checked for quality with FastQC v0.11.9 [28], and low-quality regions and TruSeq adapters were trimmed from the reads using trimmomatic v0.38 [49] using default settings. Reads were aligned against the Mus musculus reference genome (GRCm38.98) using HISAT2 v2.1.0 [50], and per-exon read counts were generated from the alignments using htseq-count [51] with the following option: “-m union -f sam -r name -t exon -i gene_id -s no”. Sequence data files were deposited in the Sequence Read Archive (SRA) (Accession: PRJNA1071120).

### 4.9. Statistical Analysis

Statistical analysis was performed using Student’s *t*-test with GraphPad Prism 7.0 software (GraphPad Software, La Jolla, CA, USA). For more than two variables, a two-way analysis of variance (ANOVA) was used followed by Tukey’s post-hoc test to determine significantly different groups. Data are presented as mean ± standard error of the mean (SEM), and *p* < 0.05 was considered statistically significant. Significance levels are indicated by * (*p* < 0.05), ** (*p* < 0.01), *** (*p* < 0.001) and **** (*p* < 0.0001).

Analysis of RNA-seq data was performed using the R software v3.6.1 and v4.0.3 environment for statistical computing and graphics [52]. Fold changes and *p*-values for differentially expressed genes were calculated using gene-wise negative binomial generalized linear models and likelihood ratio tests in edgeR [53]. Genes with *p* < 0.05 and an absolute log2 fold change > 1 were considered significantly differentially expressed.

## Figures and Tables

**Table 1:** Sequence of the oligonucleotide primers used for RT-qPCR.

Target	Species	Sense (5′-3′)	Antisense (5′-3′)
*CD206*	Human	CTACAAGGGATCGGGTTTATGGA	TTGGCATTGCCTAGTAGCGTA
*CCL2*	Human	GCCACCTTCATTCCCCAAGGG	GCTTCTTTGGGACACTTGCTGC
*CCL22*	Human	ATTACGTCCGTTACCGTCTG	TAGGCTCTTCATTGGCTCAG
*IL-1b*	Human	GGACAGGATATGGAGCAACAA	CCCAAGGCCACAGGTATTT
*GAPDH*	Human	CTCGACTTCAACAGCGACA	GTAGCCAAATTCGTTGTCATACC
*Cd163*	Mouse	GCAAAAACTGGCAGTGGG	GTCAAAATCACAGACGGAG
*Arg2*	Mouse	GAAGTGGTTAGTAGAGCTGTGTC	GGTGAGAGGTGTATTAATGTCCG
*Tnf-a*	Mouse	CTTCTGTCTACTGAACTTCGGG	CACTTGGTGGTTTGCTACGAC
*iNos*	Mouse	CAGCACAGGAAATGTTTCAGC	TAGCCAGCGTACCGGATGA
*Gapdh*	Mouse	CGTCCCGTAGACAAAATGGT	TTGATGGCAACAATCTCCAC
*GLI3*	Human	AGGGTGAATGGTATCAAGATGG	CCCACGGTTTGGTCATAGAA
*Gli3*	Mouse	CACTGGTGACCCTATGCATAATA	CTTGACTAGGGTTGTTCCTTCC

## Data Availability

RNA-sequencing data supporting reported results can be found in the National Library of Medicine (BioProject accession number: PRJNA1071120; https://www.ncbi.nlm.nih.gov/bioproject/PRJNA1071120/ (accessed on 22 October 2024).

## Supplementary Materials

The following supporting information can be downloaded at: https://www.mdpi.com/article/10.3390/ijms252313120/s1.
