# Supplementary material for: GLI3 Is Required for M2 Macrophage Polarization and M2-Mediated Waldenström Macroglobulinemia Growth and Survival
Source: Int J Mol Sci. 2024 Dec 6;25(23):13120. doi: 10.3390/ijms252313120 (PMC11641819; doi:10.3390/ijms252313120)
Supplement: Supplementary file 1 [file ijms-25-13120-s001.zip › ijms-3302580-supplementary.pdf]

Supplemental Figures

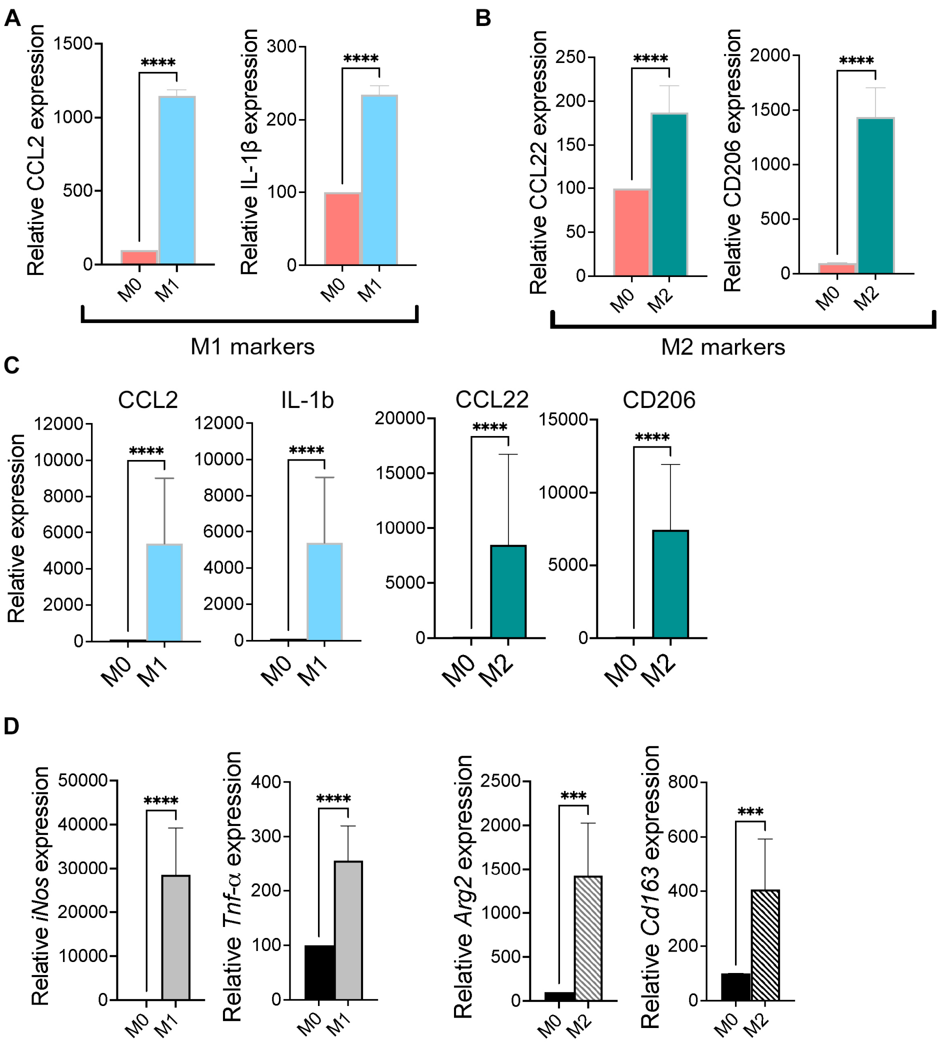

Supplemental Figure S1. Phenotypes of M0, M1 and M2 macrophages. RT-qPCR was used to confirm polarized of THP-1 cells into an (A) M1 phenotype or (B) M2 phenotype; (C) CD14+ cell polarization and (D) BMDM polarization into M1 and M2.
